# A recommendation for applied researchers to substantiate the claim that ordinal variables are the product of underlying bivariate normal distributions

**DOI:** 10.1007/s11135-016-0378-2

**Published:** 2016-07-06

**Authors:** Jarl K. Kampen, Arie Weeren

**Affiliations:** 1 Biometris, Wageningen University, Hollandseweg 1, 6706 KN Wageningen, The Netherlands; 2 StatUA, Antwerp University, Prinsstraat 13, 2000 Antwerp, Belgium

**Keywords:** Ordinal variable, Measurement scale, Polychoric correlation, Test, Acquiescence bias, Non-differentiation

## Abstract

A simulation study was carried out to study the behaviour of the polychoric correlation coefficient in data not compliant with the assumption of underlying continuous variables. Such data can produce relatively high estimated polychoric correlations (in the order of .62). Applied researchers are prone to accept these artefacts as input for elaborate modelling (e.g., structural equation models) and inferences about reality justified by sheer magnitude of the correlations. In order to prevent this questionable research practice, it is recommended that in applications of the polychoric correlation coefficient, data is tested with goodness-of-fit of the BND, that such statistic is reported in published applications, and that the polychoric correlation is not applied when the test is significant.

## Introduction

Polychoric correlations are estimates of correlations of unobserved normally distributed variables assumed to have generated observed ordinal variables. A common use of polychoric correlations is when Likert-type items are input for structural equation models. Use of the polychoric correlation coefficient is usually defended by pointing to studies where the behavior of this coefficient was tested and found adequate in data compliant with the underlying variable assumption. It has been claimed on many separate occasions that when the data generating process (DGP) of observed ordinal variables is a multivariate normal distribution, models respecting the DGP generally perform better than models not respecting it. More concretely, polychoric correlations perform better than Pearson correlations when the analysed ordinal variables are the product of multivariate underlying distributions (e.g., Holgado-Tello et al. 2010; Jöreskog and Sörbom 1988; Kukuk 1991).

Although reassuring, these and similar findings are hardly surprising because one would expect a model that corresponds to the DGP to perform better than any other model on average. Other researchers have pointed out that when the underlying distribution of continuous variables is non-normal but is skewed, leptokurtic or platykurtic, estimates of the correlation of underlying variables is biased (O’Brien and Homer 1987; Ethington 1987; Coenders et al. 1997). In cases of deviations from normality of underlying variables, other approaches than using the polychoric correlation coefficient may be more efficient (Choi et al. 2011).

Empirical researchers in practical cases are rarely able to determine whether in fact, their observed ordinal variables have corresponding underlying variables in reality (see e.g., Kampen and Swyngedouw 2000). Non-normality (e.g., skew, bimodality, etc.) of observed ordinal variables reveals nothing about the characteristics of underlying variables. The question is then, what kinds of (statistical) evidence can be taken into account to verify or falsify the hypothesis that indeed, an ordinal variable corresponds to a continuous underlying variable? A partial answer to this question can be produced by studying the behaviour of the polychoric correlation and associated test statistics in data which data generating process is *incompatible* with the underlying variable paradigm (yet fully compatible with the way data can be generated in questionnaire research). We provide such a study in the sections below.

## Verifying characteristics of the data generating process

### Definition and estimation

We consider the stochastic variables A and B measured on an ordinal scale, i.e. we assume without loss of generality that A has possible outcomes 1,…, *n*_*A*_ and similarly B has possible outcomes 1, …, *n*_*B*_. Let the probability that A = *a* and B = *b* be given by *p*_*ab*_. The basic idea behind the polychoric correlation is that there exist bivariate normally distributed variables X and Y and thresholds *τ*_*A*(*a*)_ and *τ*_*B*(*b*)_, such that A = *a* whenever *τ*_*A*(*a*−1)_ < X ≤ *τ*_*A*(*a*)_ and B = *b* whenever *τ*_*B*(*b*−1)_ < Y ≤ *τ*_*B*(*b*)_, and we assume $\tau_{{A\left( {n_{A} } \right)}}$ = $\tau_{{B\left( {n_{B} } \right)}}$ = ∞. Since the “underlying variables” X and Y are not directly observed, we can assume without loss of generality that they have unit variance, zero mean, and covariance equal to correlation cov (X, Y) = *r*. The so-called polychoric correlation coefficient $\rho$ is the estimate of the correlation *r* based on observed A and B only. In applied cases the polychoric correlation coefficient is computed by an iterative process (see e.g. Jöreskog 1994; Kukuk 1991). Denoting the bivariate standard normal distribution by *f* and sample size by *N*, a full maximum likelihood approach maximizes

$$\ell \left( {\tau_{A} ,\tau_{B} ,\rho } \right) = N\mathop \sum \limits_{a = 1}^{{n_{A} }} \mathop \sum \limits_{b = 1}^{{n_{B} }} p_{ab} { \log }\left[ {\mathop \int \limits_{{\tau_{{A\left( {a - 1} \right)}} }}^{{\tau_{A\left( a \right)} }} \mathop \int \limits_{{\tau_{{B\left( {b - 1} \right)}} }}^{{\tau_{B\left( b \right)} }} f\left( {x, y |\rho } \right)dxdy} \right]$$

and returns estimates of the threshold vectors and the correlation of the underlying variables under the assumption that X and Y have a standard normal distribution with covariance *r* = *ρ*. As stated in the introduction, when the DGP is indeed the BND, full or two-stage ML estimation produces accurate estimates of the population parameters (e.g., Maydeu-Olivares et al. 2009).

### Statistical hypothesis testing

Because ML estimation produces accurate estimates of the population parameters, it also provides an accurate representation of the DGP (Azzalini 1996). A straightforward statistical test compares expected frequencies/probabilities based on a bivariate normal distribution with given thresholds and correlation to observed frequencies/probabilities in the ordinal-by-ordinal cross table (see Pearson 1900). The expected probabilities given the assumed bivariate normal underlying distribution are

$$\hat{\pi }_{ab} = \mathop \int \limits_{{\hat{\tau }_{{A\left( {a - 1} \right)}} }}^{{\hat{\tau }_{A\left( a \right)} }} \mathop \int \limits_{{\hat{\tau }_{{\tau B\left( {b - 1} \right)}} }}^{{\hat{\tau }_{B\left( b \right)} }} f\left( {x, y |\hat{\rho }} \right)dxdy$$

and the goodness-of-fit statistic with asymptotic Chi squared distribution can be written as

$$\Delta \left( {r = \hat{\rho }} \right) = N\mathop \sum \limits_{ab} \frac{{(p_{ab} - \hat{\pi }_{ab} )^{2} }}{{\hat{\pi }_{ab} }}.$$

The bivariate normal distribution (BND) with parameter *r* can be considered a realistic data generating process of observed ordinal variables A and B when $\Delta \left( {r = \hat{\rho }} \right)$ is statistically insignificant at *df* = *n*_*A*_ *n*_*B*_−*n*_*A*_ *−n*_*B*_ = (*n*_*A*_−1)(*n*_*B*_−1)−1.

An alternative strategy is to test H_0_: *r* = 0 against the alternative H_1_: *r* = $\hat{\rho }$ by the likelihood ratio statistic:

$$\Delta \left( {r = 0} \right) = 2\left[ {\ell \left( {\tau_{A} ,\tau_{B} ,\rho } \right) - \ell \left( {\tau_{A} ,\tau_{B} ,\rho = 0} \right)} \right]$$

which has an asymptotic Chi squared distribution with *df* = 1. If Δ(*r* = 0) is significant, we reject the possibility that the data generating process was a BND with *r* = 0 in favour of the alternative hypothesis H_1_: BND with *r* = $\hat{\rho }$. Note that both $\Delta \left( {r = \hat{\rho }} \right)$ and $\Delta \left( {r = 0} \right)$ are computationally intensive because they require numerical integration of the BND. A simple alternative is therefore to employ Pearson’s goodness-of-fit Chi squared test for independence (GFXwhich significance can tentatively be accepted as evidence for H_1_: *r* ≠ 0. Tentatively, because by assuming validity of the BND as DGP the latter two reasoning procedures are instances of *abduction* (see Niiniluoto 1999) rather than statistical induction.

Summarizing, the following tests may be informative regarding the DGP of two observed ordinal variables:

1. Goodness-of-fit statistic Δ(*r* = $\hat{\rho }$) testing for goodness of fit of BND with estimated polychoric correlation coefficient in observed data;
2. Likelihood ratio statistic Δ(*r* = 0) testing H_0_: *r* = 0 against the alternative H_1_: *r* = $\hat{\rho }$
3. The GFX2, Pearson’s goodness-of-fit statistic for independence of the observed variables, tentatively corresponding to testing H_0_: *r* = 0 against the alternative H_1_: *r* ≠ 0.

A simulation study must show the adequacy of these tests.

## A simulation of data without underlying continuous association structures

### Data and method

There are many ways of producing ordinal dependent data without underlying continuous association structures. In this article, data generating processes were studied that require (much) less parameters than the bivariate normal approximation. Of course, under certain conditions, an underlying BND can be an appropriate proxy for the DGP when in reality data was generated by a different mechanism. A clear example is when data is generated by independent binomial distributions (remembering that the normal distribution can be used as approximation of the binomial). We consider therefore only cases where the DGP is neither binomial nor underlying bivariate normal. The uniform distribution is a simple example.

In this study it was assumed that the two ordinal variables were Likert-type items in a questionnaire on a five point Agree-Disagree scale (*n*_*A*_ = *n*_*B*_ = 5). It is well-known that such items in questionnaires suffer from a variety of different sources of bias (e.g., Furnham 1986). In the generation of our data, we assumed that responses could be generated by

1. Acquiescence bias, i.e. the tendency to agree with any statement regardless of its content (e.g., McClendon 1991);
2. Non-differentiation, i.e. the tendency to produce the same response to any statement regardless of its content (e.g., Krosnick and Alwin 1988);
3. Genuine reporting of level of agreement.

During simulations we specified acquiescence bias (AB) and non-differentiation (ND) to occur in 20 % of sample observations. Responses generated by genuine (dis)agreement (GA) were assumed to have independent uniform distributions (which conceptually means that there are no dominant opinions in the population). Table 1 presents the 4 probability distributions under study. Note that the number of parameters required for each scenario is considerably less than the 9 parameters needed for the BND approach. More specifically, genuine agreement (bivariate uniform distribution) requires 1 parameter, for GA and acquiescence bias 2 parameters are required, for GA and non-differentiation (which is a special case of the diagonal parameter model; see Tanner and Young 1985) the number of parameters is also 2, and for the combination GA + AB + ND a total of 3 parameters is needed.

**Table 1:** Simulated cross tables

$$\left[ {\begin{array}{ccccc} {{\text{.040}}}&{.040}&{.040}&{.040}&{.040}\\ {.040}&{{\text{.040}}}&{.040}&{.040}&{.040}\\ {.040}&{.040}&{{\text{.040}}}&{.040}&{.040}\\ {.040}&{.040}&{.040}&{{\text{.040}}}&{.040}\\ {.040}&{.040}&{.040}&{.040}&{{\text{.040}}} \end{array}} \right]$$	$$\left[ {\begin{array}{ccccc} {{\text{.132}}}&{.032}&{.032}&{.032}&{.032}\\ {.032}&{{\text{.132}}}&{.032}&{.032}&{.032}\\ {.032}&{.032}&{{\text{.032}}}&{.032}&{.032}\\ {.032}&{.032}&{.032}&{{\text{.032}}}&{.032}\\ {.032}&{.032}&{.032}&{.032}&{{\text{.032}}} \end{array}} \right]$$
Genuine agreement	GA + acquiescence bias
$$\left[ {\begin{array}{ccccc} {{\text{.072}}}&{.032}&{.032}&{.032}&{.032}\\ {.032}&{{\text{.072}}}&{.032}&{.032}&{.032}\\ {.032}&{.032}&{{\text{.072}}}&{.032}&{.032}\\ {.032}&{.032}&{.032}&{{\text{.072}}}&{.032}\\ {.032}&{.032}&{.032}&{.032}&{{\text{.072}}} \end{array}} \right]$$	$$\left[ {\begin{array}{ccccc} {{\text{.164}}}&{.024}&{.024}&{.024}&{.024}\\ {.024}&{{\text{.164}}}&{.024}&{.024}&{.024}\\ {.024}&{.024}&{{\text{.164}}}&{.024}&{.024}\\ {.024}&{.024}&{.024}&{{\text{.164}}}&{.024}\\ {.024}&{.024}&{.024}&{.024}&{{\text{.164}}} \end{array}} \right]$$
GA + non-differentiation	GA + AB + ND

Each scenario was based on *N* = 500, and was replicated 1000 times. The general procedure of the simulations was as follows:

- Simulate a sample of size *N* = 500 drawn from one of the probability distributions in Table 1;
- Estimate the thresholds and polychoric correlation coefficient using a full ML approach;
- Compute Pearson’s Chi square for independence GFX2, Δ(*r* = 0) and Δ(*r* = $\hat{\rho }$);
- Repeat steps 1–3 for 1000 times for each of the 4 probability distributions aka scenarios.

A script written in Matlab (available on request) was used to execute the simulations, applying a full maximum likelihood approach for estimation of *r* optimizing both the thresholds and the polychoric correlation coefficient.

## Results

**Fig. 1:**
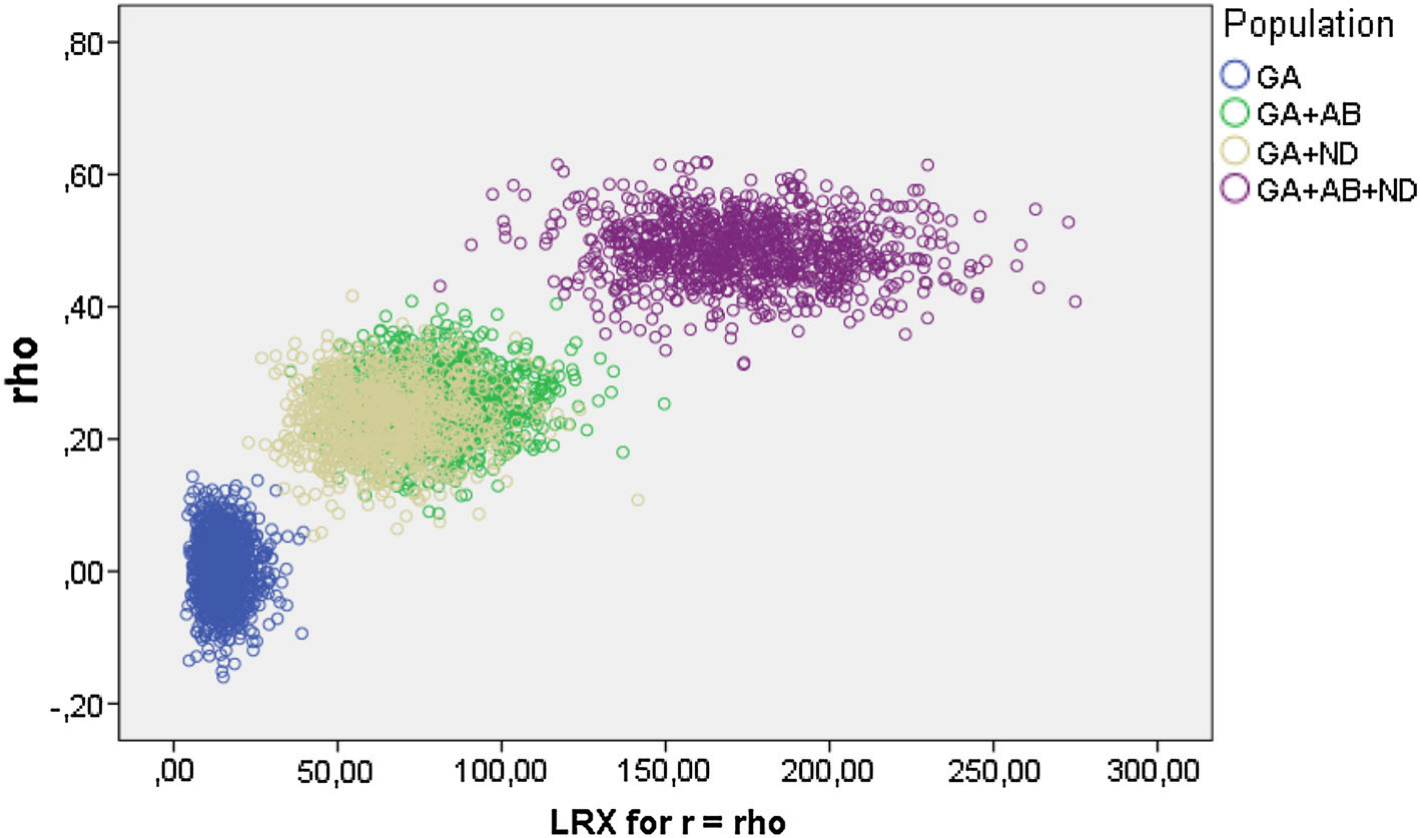
Estimated polychoric correlation and the BND fit statistic Δ(*r* = $\hat{\rho }$)

A summary of the results of the simulation study is in Table 2. It displays for each scenario, the mean estimate of the polychoric correlation coefficient, its observed standard deviation (indicative of the standard error of the parameter), and the proportion of times that the BND was accepted as data generating process conditionally on the applied test. For Δ(*r* = $\hat{\rho }$) the BND was accepted as DGP if it was insignificant at *df* = 15 (indicating good fit), for Δ(*r* = 0) the BND was accepted when it was significant at *df* = 1, and for GFX2 the BND was accepted when significant at *df* = 16. Interpreting a rejection of zero correlation or independence (GFX2 test) as an acceptance of BND reflects the pragmatic decision of applied researchers: rejecting the zero hypothesis for either test will lead to accepting estimated rho as an adequate measure of association which in itself and by itself means that BND is accepted. This reasoning is what we refer to in Sect. 2.2 as an instance of abduction rather than statistical induction. Figure 1 shows that within scenarios there is no relationship between the size of the estimated polychoric correlation and the BND fit statistic Δ(*r* = $\hat{\rho }$).

**Table 2:** Main results of the simulation study

Scenario	Mean $\hat{\rho }$ (sd)	The percentage of tests leading to the conclusion that the DGP is BND					
			Δ(*r* = 0)	GFX2	Δ(*r* = $\hat{\rho }$)	Δ(*r* = 0)	GFX2
GA	.0025 (.0499)	94.7	4.6	4.9	98.7	0.9	1.2
GA + AB	.2527 (.0526)	0.0	99.8	100.0	0.0	98.9	100.0
GA + ND	.2306 (.0542)	0.1	99.2	100.0	0.3	97.1	100.0
GA + AB + ND	.4854 (.1785)	0.0	100.0	100.0	0.0	100.0	100.0
		α = 5 %			α = 1 %

## Discussion

From the magnitude of observed $\hat{\rho }$, (with estimates as high as .62; see Fig. 1), a first warning follows that researchers must continue to be aware that combined sources of bias in questionnaire research can lead to gross overestimation of the association of variables. The study further provided evidence that in applied cases, the hypothesis that observed ordinal variables are crude measures of bivariate normally distributed (BND) underlying variables can be tested by a simple statistical test.

When two variables A and B are stochastically independent, independence will also be indicated by the size of the polychoric correlation coefficient. In these cases, the goodness-of-fit statistic for H_0_: *r* = $\hat{\rho}$ will almost invariably justify the conclusion that the two variables were generated by an underlying bivariate normal distribution (with near zero correlation). In other words, the test is uninformative when the involved variables are stochastically independent.

When A and B are stochastically dependent but dependence was not introduced by underlying bivariate normal distributions, both Pearson’s test for independence and the likelihood ratio test for H_0_: *r* = 0 are liberal in assuming that data was produced by underlying (continuous normally distributed) variables. In the scenario where genuine agreement was combined with both acquiescence bias and non-differentiation, GFX2 and the likelihood ratio test for H_0_: *r* = 0 are invariably significant, and together with the observation of fairly high estimated polychoric correlation coefficients in this scenario, this may tempt applied researchers to accept the estimated coefficient as an adequate estimate of the correlation of assumed underlying variables. Thus, when conclusions are based solely on GFX2 or Δ(*r* = 0) and the magnitude of $\hat{\rho }$, applied researchers are prone to select poor fitting models for their data that are (much) less parsimonious than the DGP. Applying a second test for goodness-of-fit of the BND with estimated polychoric correlation coefficient, that is Δ(*r* = $\hat{\rho }$) discussed above, will safeguard researchers against this fallacy in near 100 % of all cases.

Note that it is possible that the goodness-of-fit of the BND is rejected when the estimated correlation coefficient is (more or less) correct. In fact, when BND is rejected it will probably always be possible to postulate a tailor-made hybrid underlying distribution (e.g., a uniform plus an exponential distribution function) that accurately reproduces observed data and suggests a significant correlation of the underlying variables. Here the reader and the authors, like Pearson and Yule in their famous controversy (Agresti 1996), may differ in the default attitude toward ordinal variables. We claim that the fact that observed cross tables of ordinal variables can be mathematically approximated by the postulation of hybrid distributions of continuous variables does by no means imply that the assumed underlying variables exist—in fact, such underlying variables manifestly don’t exist in the four cross tables we studied because their data generating process required no such postulation. And in any case, if BND is rejected, the burden of proof that the DGP is another discretised (hybrid) continuous distribution lies with the researchers proposing such claim, and must be argued on theoretical grounds rather than goodness-of-fit of the alternative model.

## Conclusions

Determining that an animal is not a cow does not mean that it must be a horse, and the rejection of the hypothesis that the polychoric correlation coefficient equals zero cannot serve as statistical evidence of an underlying bivariate normal distribution. At the very least, that test must be seconded with a test for goodness-of-fit of the BND. If the second test is insignificant, we can at least accept the BND as an accurate *proxy* of the DGP of the two observed variables (we know of course nothing about the multivariate distributions that include other variables). This means that future researchers applying the same instrument in the same population can be expected to replicate the findings of earlier research. Whether different populations will also support the conclusion that the BND is the DGP cannot be known on the basis of that evidence, because proving that requires *sample*-*free calibration* of the two ordinal variables (Thurstone 1928; see Wright 1997: 36). Therefore, even when we may accept the BNP as DGP in isolated instances, the evidence (let alone proof) for the existence of underlying variables in all instances is weak.

If on the other hand, the second test is significant, the possibility that data was generated by an underlying bivariate *normal* distribution can be safely ruled out, but the DGP can still be any other mechanism both in- and excluding non-normal underlying variables. In such cases, the issue whether or not data was produced by underlying variables remains undecided. In other words, testing for goodness-of-fit of the BND allows for verification but not falsification of the existence of underlying variables. The inability to find falsifying evidence for underlying variables justifies doubt about the scientific value of theories based on their postulation. Furthermore, when estimated correlations are relatively high in non-normal DGPs, applied researchers are tempted to accept these artefacts as adequate input for elaborate modelling (e.g., structural equation models) and inference about reality, thereby contributing to an already wide variety of existing questionable research practices (QRP, see e.g., John et al. 2012).

## Recommendations

Researchers must continue to be aware that combined sources of bias in questionnaire research can lead to serious overestimation of the association of observed variables. We further propose that in applications of the polychoric correlation coefficient, data is tested with goodness-of-fit of the BND, that such statistic is reported in published applications, and that the polychoric correlation is not applied when the test is significant.
